# The Deubiquitinating Enzyme UCHL1 Induces Resistance to Doxorubicin in HER2+ Breast Cancer by Promoting Free Fatty Acid Synthesis

**DOI:** 10.3389/fonc.2021.629640

**Published:** 2021-02-24

**Authors:** Guangxian Lu, Jianhua Li, Leyun Ding, Chenping Wang, Lian Tang, Xin Liu, Jinhui Xu, Qin Zhou, Jiantong Sun, Wenjuan Wang, Xinyuan Ding

**Affiliations:** ^1^ Department of Pharmacy, The Affiliated Suzhou Hospital of Nanjing Medical University, Suzhou, China; ^2^ Department of General Surgery, Huashan Hospital, Fudan University, Shanghai, China; ^3^ Institute of Organ Transplantation, Fudan University, Shanghai, China; ^4^ Department of Pharmacy, Nantong Third Hospital Affiliated to Nantong University, Nantong, China; ^5^ Department of Pharmacy, Children’s Hospital of Soochow University, Soochow University, Suzhou, China

**Keywords:** breast cancer, HER2+, chemoresistance, UCHL1, free fatty acid

## Abstract

Ubiquitin C-terminal hydrolase L1 (UCHL1), which is a deubiquitinating enzyme, is known to play a role in chemoresistance in cancers. However, its potential roles and mechanisms in the chemoresistance of breast cancer (BC) remain unclear. In this study, we examined its expression in patients with BC and employed Kaplan–Meier analysis and the log-rank test for survival analyses. It was found that up-regulated UCHL1 expression was positively associated with both chemoresistance and poor prognosis, especially in patients with HER2+ BC. Moreover, UCHL1 expression was elevated in HER2+ BC cells (SK-BR-3 and BT474). Similarly, doxorubicin (DOX)-resistant BC cells (MCF-7/DOX) had higher UCHL1 levels than MCF-7 cells. CCK-8 assay showed that BC cells with higher UCHL1 levels were more resistant to DOX. Furthermore, by inhibiting UCHL1 in BC cells with elevated UCHL1 expression, we demonstrated that UCHL1 promoted DOX-resistance in BC. Mechanistically, UCHL1 probably promoted DOX-resistance of BC by up-regulating free fatty acid (FFA) synthesis, as exhibited by reduced FFA synthase expression and resurrected DOX-sensitivity upon UCHL1 inhibition. Overall, UCHL1 up-regulation is associated with DOX-resistance and poor prognosis in patients with HER2+ BC. UCHL1 induces DOX-resistance by up-regulating FFA synthesis in HER2+ BC cells. Thus, UCHL1 might be a potential clinical target for overcoming DOX resistance in patients with HER2+ BC.

## Introduction

Breast cancer (BC) is one of the most common cancers among women worldwide. Over 1.5 million women (25% of all women with cancer) are diagnosed with BC every year (1). BC is classified into three categories depending on clinical and histopathological characteristics and the expression of progesterone receptor (PR), estrogen receptor (ER), human epidermal growth factor receptor 2-related protein (HER2), and Ki67 (2). Among these, the HER2-positive (HER2+) subtype exists in about 20% of patients with BC; it is associated with high risk and is a significant poor prognostic factor in clinical therapy (3). Anthracyclines are the most active and widely employed chemotherapeutic drugs for BC (4), with a strong evidence showing positive impact on BC survival (5). Among them, doxorubicin (DOX) is one of the most common first-line chemotherapy in the clinical treatment of early and advanced BC. However, DOX resistance often occurs in clinical practice, which limits long-term treatment benefits in these patients (6).

There are multiple cellular mechanisms contributing to the development of DOX resistance. P-glycoprotein and breast cancer resistance protein (BCRP) are significant members of the adenosine triphosphate (ATP)-binding cassette (ABC) family and confer DOX resistance by increasing drug efflux, thereby reducing DOX concentrations within BC cells. This may also be mediated by overexpression of transcription-linked DNA repair pathways, topoisomerase II mutations, alterations in apoptotic signaling, and increased free fatty acid (FFA) synthesis. FFAs are critical to tumor growth, and it has been reported that these are highly expressed in BC cells. Similarly, inhibition of fatty acid synthase (FASN) inhibited tumor growth (7). The expression of FASN, which is an enzyme that catalyzes the terminal step of *de novo* synthesis of FFAs, was higher in BC cells than in normal cells (8). Overexpression of FFAs confers many advantages to tumor cells, such as BC cells. It also plays a critical role in chemoresistance acquisition (9, 10). Therefore, it is important to further understand the relationship between FFA synthesis and DOX resistance, which may help improve clinical employment of the drug.

Ubiquitination and deubiquitination are reversible post-translational modifications that depend on ubiquitin ligases and deubiquitinating enzymes (DUBs) (11). DUB-induced cleavage of ubiquitin chains from substrate proteins can play an important role in different cellular processes (12). The impact of DUBs on chemoresistance in cancers has been reported. Our previous studies have demonstrated that ubiquitin C-terminal hydrolase L1 (UCHL1), which is a member of the DUB family, could promote resistance to pemetrexed in non-small cell lung cancer (NSCLC) by up-regulating thymidylate synthase (13). However, the role of UCHL1 in DOX resistance in BC remains unclear.

Thus, our findings demonstrate that UCHL1 is highly expressed in BC tissues and cells, especially in the HER2+ BC type, and is positively associated with poor prognosis and DOX resistance. We also show that UCHL1 induces DOX resistance in HER2+ BC cells by promoting FFA synthesis. These data demonstrate that UCHL1 may be a potential target to overcome DOX resistance in clinical therapy of patients with HER2+ BC.

## Materials and Methods

### Patients and BC Specimens

Fifty-four primary BC tissues were obtained from patients in the Affiliated Suzhou Hospital of Nanjing Medical University (Suzhou, China) between January 2012 and March 2014. The patients with pathologically confirmed BC disease had not undergone chemotherapy or radiotherapy; they underwent postoperative chemotherapy containing DOX according to the BC guidelines provided by the National Comprehensive Cancer Network (NCCN). All patients provided informed consent for specimen collection and analysis. According to the Response Evaluation Criteria in Solid Tumors (RECIST, version 1.1), the patients with BC and their specimens were considered as “chemosensitive” if they had complete or partial response or “chemoresistant” if they had progressive disease (13). All experimental protocols were approved by the ethics committee of the Affiliated Soochow Hospital of Nanjing Medical University and were in accordance with the principles of the Declaration of Helsinki.

### Immunohistochemistry

Immunohistochemistry (IHC) was performed using a standard immunoperoxidase staining procedure to test the expression of UCHL1 in paraffin-embedded BC specimens. The primary antibody was a rabbit anti-human UCHL1 antibody (1:400; Cell Signaling Technology, Danvers, Massachusetts, USA), and secondary staining was performed using an anti-rabbit secondary antibody and the DAKO ChemMate TM Envision TM Detection Kit (DAKO A/S, Denmark). Positive staining for UCHL1 (brown) was largely localized in the cytoplasm. IHC staining was scored using a H-score system dependent on both the staining intensity and the percentage of UCHL1-positive tumor cells. The staining intensity was scored as negative (0), weak (1+), moderate (2+), and strong (3+). The H-score was calculated using the following formula: 1 × (percentage of cells stained weakly [1+]) + 2 × (percentage of cells stained moderately [2+]) + 3 × (percentage of cells stained intensely [3+]), with overall scores ranging from 0 to 300 (13). For the cohort dichotomization into two subgroups based on chemotherapy response, UCHL1 expression in patients with BC was assessed by the R statistical environment employing the “survival ROC” package to determine the optimal cut-off value for defining high or low UCHL1 expression.

### Validation Using Human Databases

The detailed procedure has been described previously (14). Clinical data from patients with BC were obtained from the Kaplan Meier-plotter database, as a publicly acceptable BC database (https://kmplot.com/analysis/, 201387_s_at). Kaplan–Meier survival analysis was conducted in different subtype patients with BC.

### Establishment of DOX-Resistant BC Cells

MCF7 cells were purchased from the American Type Culture Collection (ATCC, USA). DOX-resistant (DOX-R) cell lines were named MCF7/DOX and were established by exposing the parental cell lines to stepwise increment of DOX (Haizheng, Zhejiang, China) at a 50% inhibitory concentration (IC50) for 6 months. The DOX-R BC cells were verified to have acquired stable resistance and were employed for subsequent experiments.

### Cell Proliferation

Cell proliferation was observed using the Cell Counting Kit-8 (CCK-8; Dojindo, Kumamoto, Japan), which was in accordance to the manufacturer’s instructions.

### Western Blot Analysis

A detailed procedure was reported in a previous study (15). Primary anti-human antibodies against UCHL1, FASN, acetyl-CoA carboxylase, and GAPDH were purchased from Cell Signaling Technology.

### Real-Time Quantitative PCR

Detailed procedures have been previously reported (14). The primer sequences employed for the PCR analysis are listed in **Table S1**. All primers were synthesized by Sangon Biotech (Shanghai, China).

### Cell Fatty Acid Extraction and Analysis

Fatty acid (FA) extraction: 1 ml 5% H_2_SO_4_/methyl alcohol was added to tubes containing cell pellets (2 × 10^6^) and supplemented with 100 μg nonadecanoic acid methyl ester as a confidential standard. N_2_ was added to exclude air from the tubes, which were heated at 80°C for 90 min. After cooling the tubes at 4°C for 10 min, 1.5 ml double distilled H_2_O and 1 ml hexane were added into the tubes. Vortexed mixtures were centrifuged at 2,000 rpm for 2 min, and the upper phase was used for FA analysis.

FA analysis: Gas chromatography–mass spectrometry (GC–MS, 7890A-5975C, Agilent Technologies, Santa Clara, CA, USA) was employed to measure FA types (containing fatty acids C14:0, C16:0, C16:1, C18:0, and C18:1) and contents. FA concentrations were determined based on the confidential standard (16).

### Flow Cytometry

Flow cytometry (FCM) was performed using an Annexin V/PI assay kit (Thermo Fisher, Massachusetts, USA) according to the manufacturer’s protocol and analyzed using a FACS Calibur flow cytometer (BD Biosciences, San Jose, CA, USA).

### Statistical Analysis

Statistical analyses were performed using IBM SPSS software (version 20) and GraphPad Prism software (version 7). All measurement data were shown as mean ± standard error. The Mann–Whitney test and analysis of variance were employed to compare continuous variables. Relationships between the UCHL1 expression and clinicopathological characteristics were assessed using the χ^2^ test or Fisher’s exact test. Survival curves were created using the Kaplan–Meier method and compared using the log-rank test. Differences were considered statistically significant at a *p*-value of <0.05.

## Results

### Higher UCHL1 Expression Was Associated With DOX Resistance in Patients With BC

**Figure 1:**
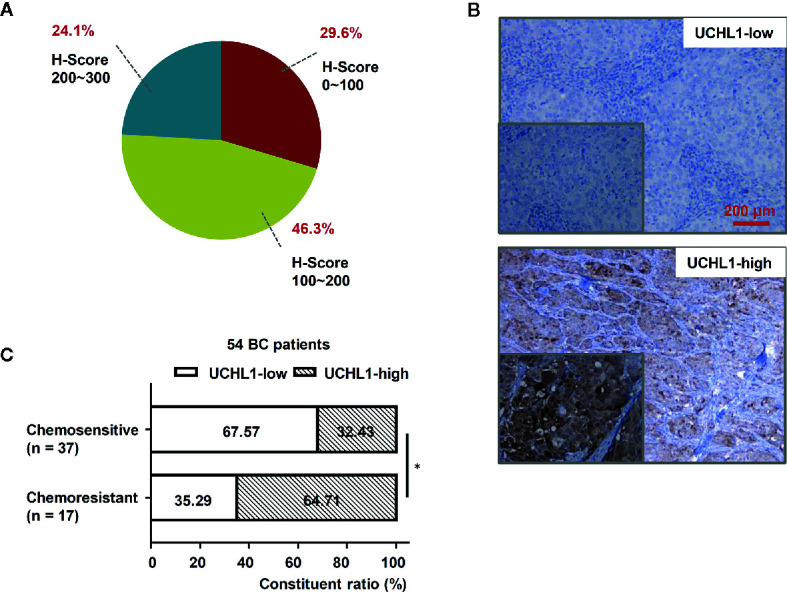
High UCHL1 expression was associated with chemoresistance in patients with BC. **(A)** The scores (ranged from 0 to 300) of IHC staining of UCHL1 expression in 54 BC samples were calculated and its proportions are shown. **(B)** Representative images showing both UCHL1-high and UCHL1-low expression were presented (red bar: 200 μm). Comparison of chemoresistant status in 54 BC patients with different levels of UCHL1 expression **(C)**. Statistical analysis was performed with χ^2^ test. *p < 0.05.

To explore the role of UCHL1 in DOX resistance in BC, we evaluated IHC and showed that UCHL1 was differentially expressed in 54 patients with BC (**Figure 1A**), with an IHC score cut-off value of 135, which was employed to classify UCHL1-high or UCHL1-low expression (**Figure 1B**). Moreover, the patients were classified as chemosensitive (37 patients) or chemoresistant (17 patients), depending on their responses to clinical treatment based on DOX. Patients with high UCHL1 expression showed a higher rate of resistance to clinical chemotherapy (**Figure 1C**), although UCHL1 expression was not significantly associated with any other clinicopathological characteristics (**Table 1**). These results suggest that UCHL1 is a potential marker in clinical BC therapy and is positively associated with chemoresistance.

**Table 1:** The association between UCHL1 expression and clinical pathologic characteristics in 54 patients with BC.

Clinical pathologic characteristics		Case No.	UCHL1 expression		*p*
			low	high
Total cases		54	31	23	
Age (years)					
	<60	25	12	13	0.1943
	≥60	29	19	10	
Grade					
	I~II	21	13	8	0.8150
	III	29	17	12	
	unknown	4	1	3	
Regional lymph node invasion					
	N0~N1	32	21	11	0.0703
	N2~N3	20	8	12	
	Nx	2	2	0	
Chemotherapeutics (DOX-based)					
	Chemosensitive	37	25	12	0.0259^*^
	Chemoresistant	17	6	11	

Analysis by χ^2^ test or Fisher’s exact test, *p < 0.05 value is set for highly significant difference.

### UCHL1 Expression Was Probably Positively Associated With Poor Prognosis of Patients With BC

**Figure 2:**
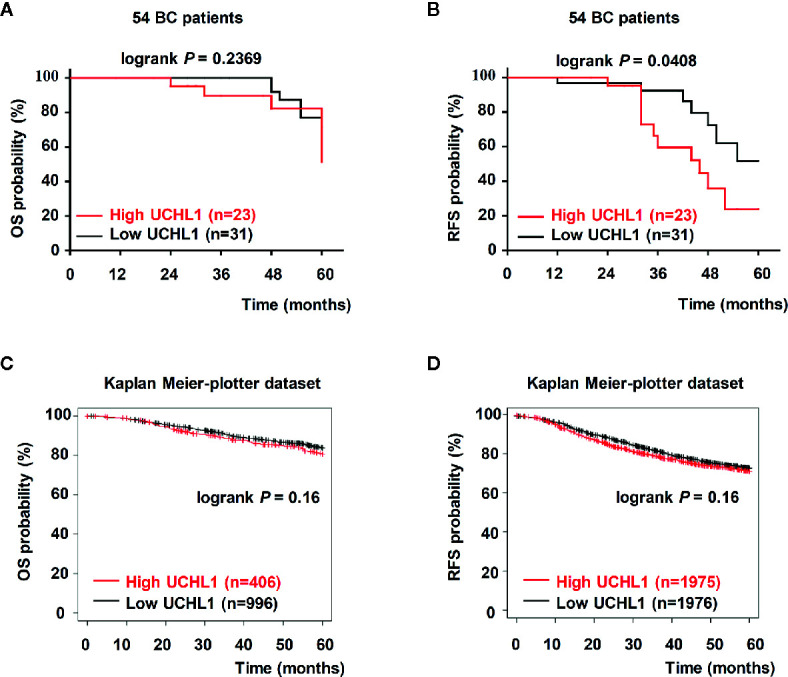
Upregulation of UCHL1 correlated with poor prognosis in patients with BC. **(A, B)** Kaplan–Meier analysis of overall survival (OS) and recurrence-free survival (RFS) in 54 patients with BC based on UCHL1 expression are indicated. OS and RFS were determined according to UCHL1 expression in BC samples from the public Kaplan–Meier-plotter dataset **(C, D)**. Statistical analysis was carried out by Log-rank test.

Kaplan–Meier analysis showed that in 54 patients with BC, high UCHL1 levels had no correlation with overall survival (OS, **Figure 2A**) but correlated with poor recurrence-free survival (RFS, **Figure 2B**). Based on cases from the publicly acceptable databases (Kaplan–Meier-plotter dataset), we further ensured that there was no correlation between UCHL1 levels with OS (**Figure 2C**) and RFS (**Figure 2D**) in patients with BC. All these findings demonstrated that high UCHL1 expression was probably positively associated with poor prognosis in patients with BC.

### UCHL1 Was Overexpressed and Associated With DOX-Resistance in HER2+ BC Cells

**Figure 3:**
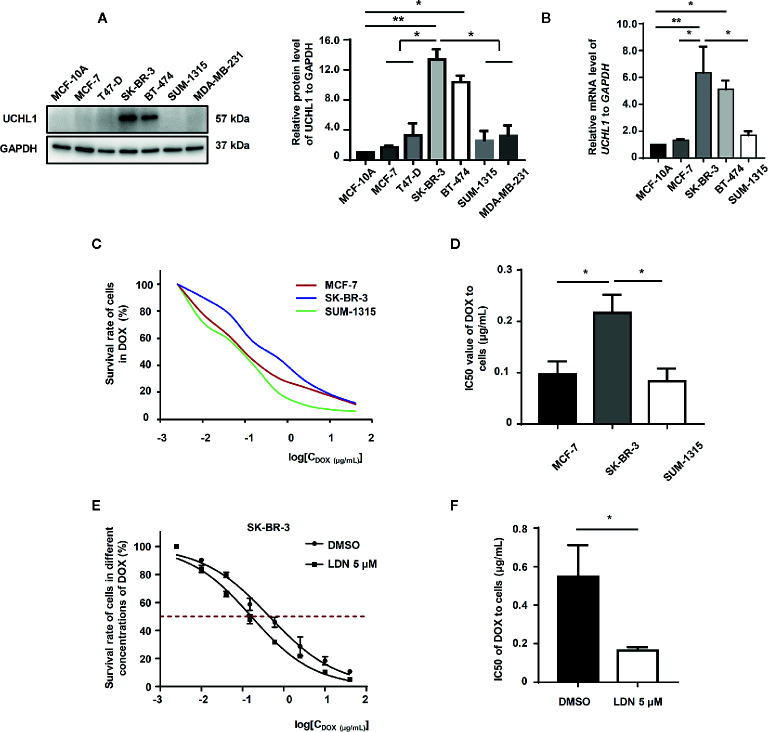
UCHL1 level and its role in BC cells. Western blot **(A)** and real-time PCR **(B)** analysis of UCHL1 levels in BC cells are shown and were analyzed by Mann-Whitney test (n = 3). Cell viability curves of BC cells treated with DOX, as evaluated by CCK8 assay **(C)**. The IC50 values, as analyzed by Mann–Whitney test (n = 3) **(D)**. Cell viability curves and IC50 values of SK-BR-3 cells treated with DOX in the presence of 5 μM LDN or DMSO are shown (n = 3) **(E, F)**. ^*^ *p* < 0.05, ^**^ *p* < 0.01.

We examined UCHL1 expression in normal mammary epithelial cells (MCF-10A) and some subtypes of BC cells (ER+ HER2−: MCF-7 and T47D; HER2+: SK-BR-3 and BT474; ER− HER2−: SUM-1315 and MDA-MB-231). Compared to HER2− BC cells, the mRNA and protein levels of UCHL1 in HER2+ BC cells (SK-BR-3 and BT474) were significantly increased (**Figures 3A, B**). By detecting IC50 values of subtype representative cell lines in the presence of DOX using CCK-8, we discovered that HER2+ BC cells had a higher survival rate at different concentrations of DOX (**Figure 3C**), with a significantly increased IC50 value (**Figure 3D**). LDN-57444 (LDN), which did not significantly affect cell proliferation at 5 µM, inhibited UCHL1 (13) in SK-BR-3 cells. Specifically, the IC50 values of SK-BR-3 cells sharply decreased when treated together with LDN (**Figures 3E, F**). Thus, we confirmed that the UCHL1 level was high and positively associated with DOX-resistance in HER2+ BC cells.

### UCHL1 Induced DOX-Resistance in BC Cells by Promoting FFA Synthesis

**Figure 4:**
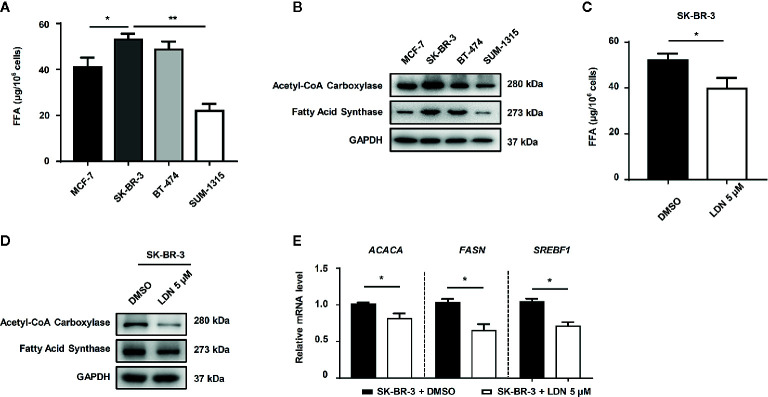
The role of UCHL1 in fatty acid synthesis in BC cells. The level of fatty acids in different subtype BC cells **(A)** and SK-BR-3 cells in the presence of 5 μM LDN or DMSO **(C)**. Western blot **(B, D)** and real-time PCR **(E)** analysis of lipogenesis-associated proteins in BC cells are shown and were analyzed by Mann–Whitney test (n = 3). *p < 0.05, **p < 0.01.

Furthermore, we explored the relationship between UCHL1 and FFA synthesis in DOX-resistance in BC cells. The level of FFAs in HER2+ BC cells was higher than that in HER2− BC cells (**Figure 4A**). The lipogenesis-associated proteins, such as acetyl-CoA carboxylase and fatty acid synthase, were up-regulated in HER2+ BC cells (**Figure 4B**). Furthermore, HER2+ BC cells (SK-BR-3), which inhibited UCHL1 activity by LDN, showed lower FFA levels (**Figure 4C**). The lipogenesis-associated proteins and genes (*ACACA*, *FASN*, and *SREBF1*) were downregulated in SK-BR-3 cells with UCHL1 inhibition (**Figures 4D, E**).

**Figure 5:**
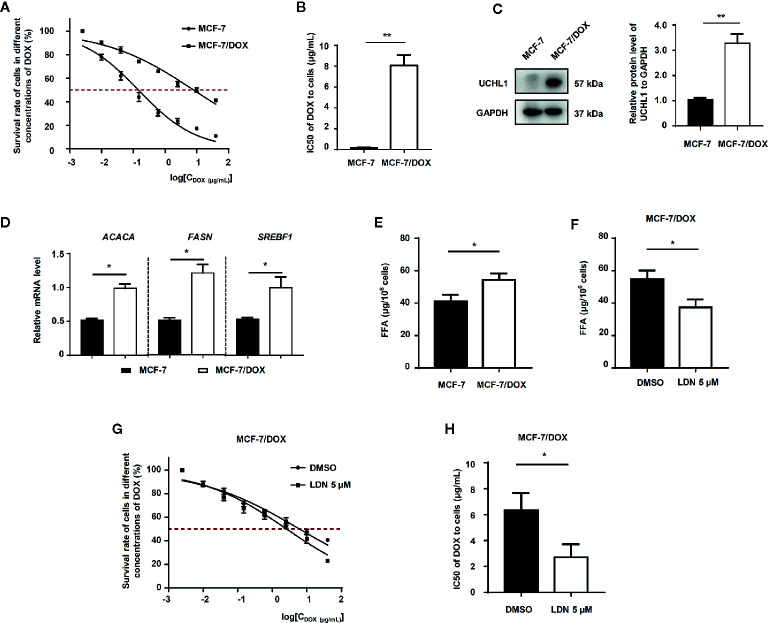
The role of UCHL1 in fatty acid synthesis in BC cells. **(A, B)** Cell viability curves and IC50 values of MCF-7 and DOX-resistant MCF-7/DOX cells treated with DOX are shown (n = 3). Western blot analysis of UCHL1 **(C)** and real-time PCR analysis of lipogenesis-associated genes **(D)** in MCF-7 and MCF-7/DOX cells are shown and were analyzed by Mann-Whitney test (n = 3). The level of fatty acids in MCF-7 and MCF-7/DOX cells **(E)** and MCF-7/DOX cells in the presence of 5 μM LDN or DMSO **(F)**. **(G, H)** Cell viability curves and IC50 values of MCF-7/DOX cells treated with DOX in the presence of 5 μM LDN or DMSO are shown (n = 3). ^*^ *p* < 0.05, ^**^ *p* < 0.01.

To further demonstrate the role of UCHL1 in FFA synthesis in DOX-resistance in other subtypes of BC cells, we established DOX-resistant cells (MCF7/DOX) with a resistance index (RI) of 51.6 (**Figures 5A, B**). The UCHL1 levels in MCF7/DOX were higher than the parental cells (**Figure 5C**). Moreover, the mRNA levels of lipogenesis-associated genes and the levels of FFAs in MCF7/DOX cells were significantly higher compared to the parental cells (**Figures 5D, E**). Based on these results, we then performed UCHL1 inhibition with LDN in MCF7/DOX cells. The FFA level in MCF7/DOX cells with UCHL1 inhibition was significantly reduced (**Figure 5F**), and its IC50 value to DOX was lower than that of MCF7/DOX cells with DMSO (**Figures 5G, H**). These findings suggested that UCHL1 was positively related to the FFA level in HER2+ BC cells and probably induced DOX resistance by promoting FFA synthesis.

### UCHL1 Was Positively Associated With Poor Clinical Prognosis of Patients With HER+ BC

**Figure 6:**
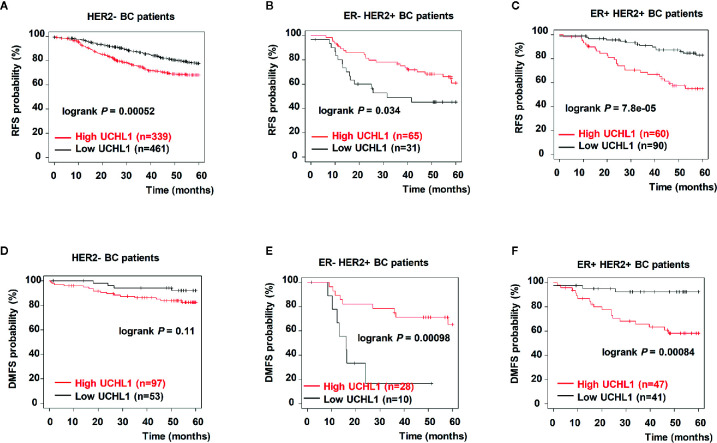
Upregulation of UCHL1 correlated with poor prognosis in patients with different subtypes of BC. Kaplan–Meier analysis of RFS and distant metastasis free survival (DMFS) in 54 patients with BC based on UCHL1 expression and different genetic typing are indicated **(A–C)**. RFS and DMFS, as determined according to UCHL1 expression and genetic typing in BC samples from the Kaplan–Meier-plotter dataset **(D–F)**. Statistical analysis was carried out by Log-rank test.

To further investigate the correlation between UCHL1 levels and poor prognosis in patients with BC, HER2+ and HER2− BC samples from the Kaplan–Meier-plotter dataset were reanalyzed. As expected, we found that ER+ HER2+ patients with high UCHL1 expression had poorer RFS (**Figures 6B, C**) and distant metastasis-free survival (DMFS, **Figures 6E, F**). However, although UCHL1 expression correlated with RFS in patients with HER2− BC, it did not correlate with DMFS (**Figures 6A, D**). These findings suggested that UCHL1 was positively associated with poor prognosis in patients with HER2+ BC.

## Discussion

**Figure 7:**
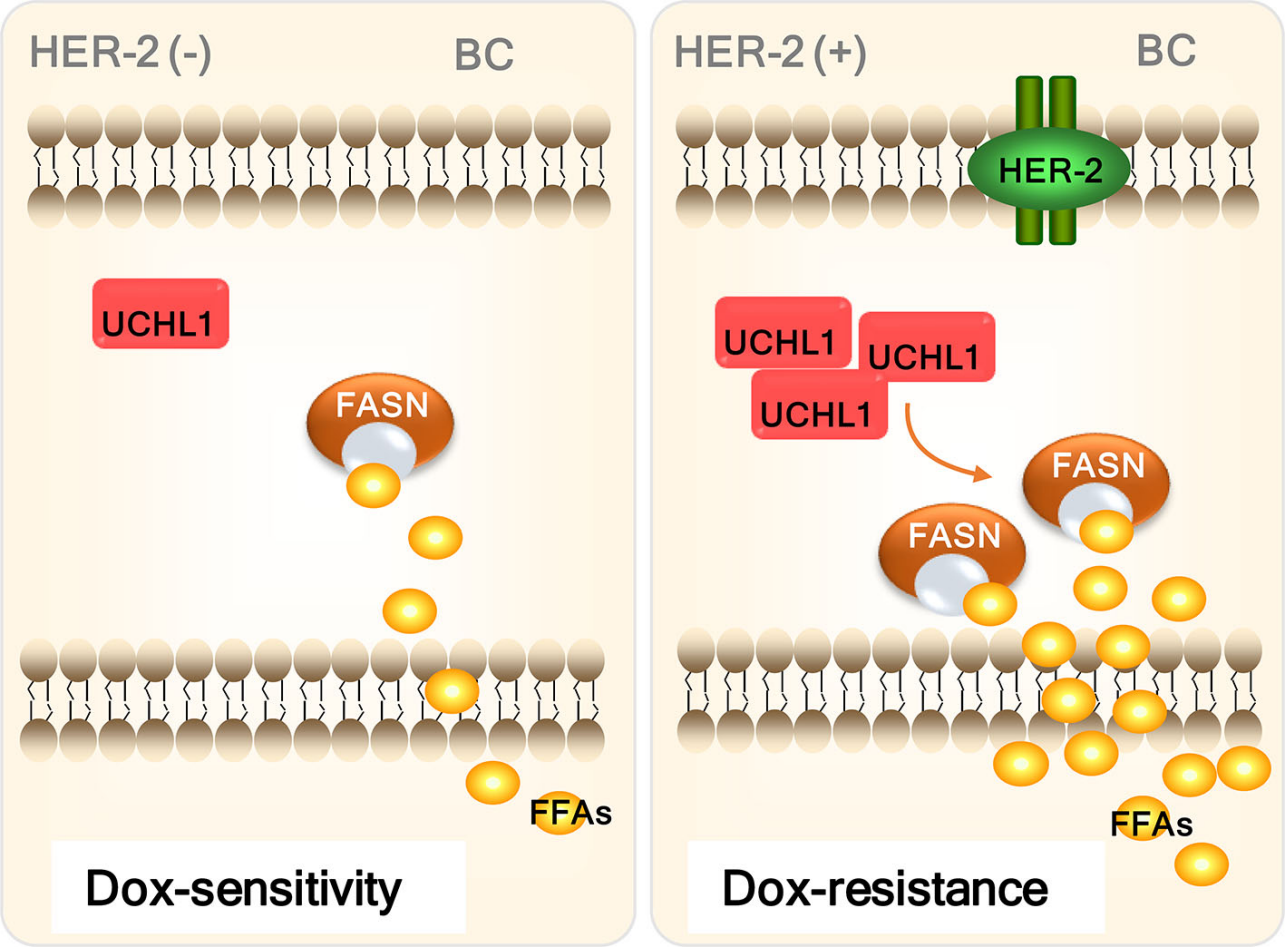
Schematic diagram of the mechanism by which UCHL1 confers DOX-resistance in BC.

Systemic chemotherapy has been the core treatment strategy for both postoperative and non-postoperative BC for decades, and it is still a critical component of treatment regimens. Chemotherapeutic agents, especially anthracyclines, are useful for most patients with BC, including patients with HER2+ BC (17). DOX, as an anthracycline drug, is one of the first-line chemotherapy drugs for patients with BC (18). However, DOX resistance still hinders the treatment of patients with BC (19). We found that UCHL1 expression was positively associated with poor prognosis and DOX-resistance in patients with BC. Interestingly, UCHL1 was highly expressed in HER2+ BC cells and DOX-resistant BC cells, while UCHL1 inhibition significantly improved the HER2+ cell sensitivity to DOX. Moreover, we discovered that UCHL1 conferred DOX-resistance in patients with BC by promoting FFA synthesis (**Figure 7**).

HER2, which is a protooncogene encoding epidermal growth factor receptor with tyrosine kinase activity, is amplified in 15–20% of invasive BC. HER2 amplification is a poor prognostic factor related to a high rate of mortality and recurrence. Similarly, it is also a predictive factor associated with response to anthracycline-based chemotherapies in patients with BC (20). It has been reported that increased HER2 could promote glycolysis *via* activation of the Akt/mTOR/HIF-1*α* axis, thus, inducing tamoxifen resistance in BC (21). DUBs are critical components of the ubiquitin-proteasome system (UPS). The basic role of DUBs is the characteristic removal of ubiquitin from substrates. Altered DUB activity is related to multiple cancers (22). Previous studies showed that ubiquitin-specific peptidase 18 (USP18) could promote HER2+ BC progression by up-regulating EGFR and activating the Akt/Skp2 pathway (23). UCHL1, which is a member of DUBs, is associated with chemoresistance in many cancers. For example, the level of UCHL1 is negatively related to cisplatin resistance in ovarian cancer, and knockdown of UCHL1 promotes cisplatin resistance (24). However, the role of UCHL1 in HER2+ BC is poorly researched. Our study found that high UCHL1 expression was associated with poor chemosensitivity of HER2+ BC cells, and Kaplan–Meier analysis based on the public dataset showed that patients with high UCHL1 expression, especially in ER+ HER2+ BC, had poorer RFS and DMFS, without any significant correlation with OS. The correlation between UCHL1 expression and RFS in patients with ER- HER2+ BC was opposite, probably owing to the limited number of patients with this subtype of BC, or the effective targeted drug (trastuzumab) for anti-HER2 treatment.

Chemoresistance in tumors can be caused by numerous factors related to either acquisition or *de novo* mechanisms (25). Accumulating evidence has demonstrated that DUBs play critical roles in chemoresistance (26). Nevertheless, whether UCHL1 plays a critical role in DOX-resistance in BC remains unclear. Our findings showed that the level of UCHL1 was positively related to chemoresistance in patients with HER2+ BC. Although it is difficult to discriminate between intrinsic resistance and acquired resistance using human specimens, UCHL1 induces acquired resistance in DOX-resistant cells, which indicates that UCHL1 plays an important role in acquired resistance.

There are various mechanisms of DOX resistance that can affect the treatment of patients with BC. Overexpression of ABC-transporters, topoisomerase II mutations, and inhibition of cell apoptosis are known to mediate DOX-resistance (27, 28). The increase in FFA utilization can also significantly promote DOX resistance (29). Significantly, the mechanisms by which UCHL1 induces chemoresistance in some cancers, such as pediatric high-grade gliomas and hepatoma cells, have been reported previously (30, 31). However, the mechanism by which UCHL1 induces DOX resistance in BC is still unknown. Here, we found that the FFA levels were increased and expression of both lipogenesis-associated proteins and genes (FASN) were higher in HER2+ BC cells with highly expressed UCHL1 than in HER2− BC cells with low-expressed UCHL1. Furthermore, the FFA levels were decreased in HER2+ BC cells with UCHL1 inhibition, and the IC50 value with DOX treatment was also decreased. It was previously reported that FFA could maintain the proliferation and aggressiveness in BC cells by activating both the ER*α* and mTOR pathways (32). Elevated FASN expression had also been shown to promote cell survival and contribute to DOX resistance in BC (33). The findings indicated that overexpression of UCHL1 promoted FFA synthesis to induce DOX resistance in HER2+ BC cells, and combination of UCHL1 inhibition and FASN inhibition could improve the DOX resistance.

Although we have found a correlation between UCHL1 and FFA synthesis in DOX-resistance of BC, further studies are still needed to explore the downstream target proteins to confirm our findings and provide further evidence supporting the role of UCHL1 in the chemoresistance of BC. *In vivo* experiments are also needed to support our findings and support the possibility of UCHL1 inhibition in overcoming DOX-resistance.

Our present findings revealed that DOX-resistance in HER2+ BC cells relied on UCHL1 expression. Moreover, UCHL1 induced DOX-resistance in HER2+ BC cells by promoting FFA synthesis. Therefore, UCHL1 plays an important role in the DOX-resistance of HER2+ BC cells and may become a promising therapeutic target for overcoming DOX-resistance in patients with HER2+ BC.

## Data Availability Statement

The datasets presented in this study can be found in online repositories. The names of the repository/repositories and accession number(s) can be found in the article/**Supplementary Material**.

## Ethics Statement

The studies involving human participants were reviewed and approved by the Medical Ethics Committee of Suzhou Municipal Hospital. The patients/participants provided their written informed consent to participate in this study.

## Author Contributions

XD and WW conceived and designed the study. GL, JL, LD, CW, LT, and XL performed the experiments and analyzed the data. GL and XD wrote the manuscript. GL, JX, QZ, and JS participated in data collection of clinical parameters. All authors contributed to the article and approved the submitted version.

## Funding

This work was supported by grants from the National Natural Science Foundation of China (No. 81902320), Wu Jieping Medical Foundation (No. 320.6750.2020-04-37), Foundation of Jiangsu Pharmaceutical Association (No. H202052), Suzhou Science and Technology Development Plan Project (No. SYSD2020188, SYSD2019183 and SYSD2019189) and the Program of Suzhou Municipal Health and Health Committee (No. KJXW2017033). The funders were not involved in study design, data collection, data analysis, nor writing of the paper.

## Conflict of Interest

The authors declare that the research was conducted in the absence of any commercial or financial relationships that could be construed as a potential conflict of interest.

## References

[1] Sun YS Zhao Z Yang ZN Xu F Lu HJ Zhu ZY. Risk factors and preventions of breast cancer. Int J Biol Sci (2017) 13:1387–97. 10.7150/ijbs.21635 PMC5715522 29209143

[2] Palomeras S Ruiz-Martínez S Puig T. Targeting Breast cancer stem cells to overcome treatment resistance. Molecules (2018) 23:2193. 10.3390/molecules23092193 PMC6225226 30200262

[3] Loibl S Gianni L. HER2-positive breast cancer. Lancet (2017) 389:2415–29. 10.1016/s0140-6736(16)32417-5 27939064

[4] Moreno-Aspitia A Perez EA. Treatment options for breast cancer resistant to anthracycline and taxane. Mayo Clin Proc (2009) 84:533–45. 10.1016/s0025-6196(11)60585-5 PMC2688627 19483170

[5] Shah AN Gradishar WJ. Adjuvant Anthracyclines in Breast Cancer: What Is Their Role? Oncologist (2018) 23:1153–61. 10.1634/theoncologist.2017-0672 PMC6263120 30120159

[6] Lovitt CJ Shelper TB Avery VM. Doxorubicin resistance in breast cancer cells is mediated by extracellular matrix proteins. BMC Cancer (2018) 18:41. 10.1186/s12885-017-3953-6 29304770 PMC5756400

[7] Kleinfeld AM Okada C. Free fatty acid release from human breast cancer tissue inhibits cytotoxic T-lymphocyte-mediated killing. J Lipid Res (2005) 46:1983–90. 10.1194/jlr.M500151-JLR200 15961785

[8] Wang X Jiang B Lv H Liang Y Ma X. Vitisin B as a novel fatty acid synthase inhibitor induces human breast cancer cells apoptosis. Am J Transl Res (2019) 11:5096–104. PMC6731432 31497225

[9] Giró-Perafita A Rabionet M Planas M Feliu L Ciurana J Ruiz-Martínez S. EGCG-Derivative G28 shows high efficacy inhibiting the mammosphere-forming capacity of sensitive and resistant TNBC models. Molecules (2019) 24:1027. 10.3390/molecules24061027 PMC6471537 30875891

[10] Liu H Liu Y Zhang JT. A new mechanism of drug resistance in breast cancer cells: fatty acid synthase overexpression-mediated palmitate overproduction. Mol Cancer Ther (2008) 7:263–70. 10.1158/1535-7163.mct-07-0445 18281512

[11] Wang WJ Li QQ Xu JD Cao XX Li HX Tang F. Over-expression of ubiquitin carboxy terminal hydrolase-L1 induces apoptosis in breast cancer cells. Int J Oncol (2008) 33:1037–45. 10.3892/ijo_00000092 PMC2628585 18949367

[12] Ning F Xin H Liu J Lv C Xu X Wang M. Structure and function of USP5: Insight into physiological and pathophysiological roles. Pharmacol Res (2020) 157:104557. 10.1016/j.phrs.2019.104557 31756387

[13] Ding X Gu Y Jin M Guo X Xue S Tan C. The deubiquitinating enzyme UCHL1 promotes resistance to pemetrexed in non-small cell lung cancer by upregulating thymidylate synthase. Theranostics (2020) 10:6048–60. 10.7150/thno.42096 PMC7255002 32483437

[14] Gu Y Lv F Xue M Chen K Cheng C Ding X. The deubiquitinating enzyme UCHL1 is a favorable prognostic marker in neuroblastoma as it promotes neuronal differentiation. J Exp Clin Cancer Res (2018) 37:258. 10.1186/s13046-018-0931-z 30359286 PMC6203192

[15] Wang W Xiong Y Ding X Wang L Zhao Y Fei Y. Cathepsin L activated by mutant p53 and Egr-1 promotes ionizing radiation-induced EMT in human NSCLC. J Exp Clin Cancer Res (2019) 38:61. 10.1186/s13046-019-1054-x 30732622 PMC6367810

[16] Wang X He S Gu Y Wang Q Chu X Jin M. Fatty acid receptor GPR120 promotes breast cancer chemoresistance by upregulating ABC transporters expression and fatty acid synthesis. EBioMedicine (2019) 40:251–62. 10.1016/j.ebiom.2018.12.037 PMC6413582 30738829

[17] Ma F Ouyang Q Li W Jiang Z Tong Z Liu Y. Pyrotinib or Lapatinib combined with capecitabine in HER2-Positive metastatic breast cancer with prior taxanes, anthracyclines, and/or trastuzumab: a randomized, phase II study. J Clin Oncol (2019) 37:2610–9. 10.1200/jco.19.00108 31430226

[18] Zhang Y Xia F Zhang F Cui Y Wang Q Liu H. miR-135b-5p enhances doxorubicin-sensitivity of breast cancer cells through targeting anterior gradient 2. J Exp Clin Cancer Res (2019) 38:26. 10.1186/s13046-019-1024-3 30665445 PMC6341729

[19] Ji X Lu Y Tian H Meng X Wei M Cho WC. Chemoresistance mechanisms of breast cancer and their countermeasures. BioMed Pharmacother (2019) 114:108800. 10.1016/j.biopha.2019.108800 30921705

[20] Jerusalem G Lancellotti P Kim SB. HER2+ breast cancer treatment and cardiotoxicity: monitoring and management. Breast Cancer Res Treat (2019) 177:237–50. 10.1007/s10549-019-05303-y PMC6661020 31165940

[21] Gandhi N Das GM. Metabolic Reprogramming in breast cancer and its therapeutic implications. Cells (2019) 8:89. 10.3390/cells8020089 PMC6406734 30691108

[22] Farshi P Deshmukh RR Nwankwo JO Arkwright RT Cvek B Liu J. Deubiquitinases (DUBs) and DUB inhibitors: a patent review. Expert Opin Ther Pat (2015) 25:1191–208. 10.1517/13543776.2015.1056737 PMC4834700 26077642

[23] Tan Y Zhou G Wang X Chen W Gao H. USP18 promotes breast cancer growth by upregulating EGFR and activating the AKT/Skp2 pathway. Int J Oncol (2018) 53:371–83. 10.3892/ijo.2018.4387 29749454

[24] Jin C Yu W Lou X Zhou F Han X Zhao N. UCHL1 is a putative tumor suppressor in ovarian cancer cells and contributes to cisplatin resistance. J Cancer (2013) 4:662–70. 10.7150/jca.6641 PMC3805994 24155778

[25] Holohan C Van Schaeybroeck S Longley DB Johnston PG. Cancer drug resistance: an evolving paradigm. Nat Rev Cancer (2013) 13:714–26. 10.1038/nrc3599 24060863

[26] Qin T Li B Feng X Fan S Liu L Liu D. Abnormally elevated USP37 expression in breast cancer stem cells regulates stemness, epithelial-mesenchymal transition and cisplatin sensitivity. J Exp Clin Cancer Res (2018) 37:287. 10.1186/s13046-018-0934-9 30482232 PMC6258492

[27] Munkácsy G Abdul-Ghani R Mihály Z Tegze B Tchernitsa O Surowiak P. PSMB7 is associated with anthracycline resistance and is a prognostic biomarker in breast cancer. Br J Cancer (2010) 102:361–8. 10.1038/sj.bjc.6605478 PMC2816652 20010949

[28] Pilco-Ferreto N Calaf GM. Influence of doxorubicin on apoptosis and oxidative stress in breast cancer cell lines. Int J Oncol (2016) 49:753–62. 10.3892/ijo.2016.3558 27278553

[29] Vrignaud P Robert J. Free fatty acid uptake is increased in doxorubicin-resistant rat glioblastoma cells. Biochim Biophys Acta (1987) 902:149–53. 10.1016/0005-2736(87)90146-5 2886153

[30] Sanchez-Diaz PC Chang JC Moses ES Dao T Chen Y Hung JY. Ubiquitin carboxyl-terminal esterase L1 (UCHL1) is associated with stem-like cancer cell functions in pediatric high-grade glioma. PLoS One (2017) 12:e0176879. 10.1371/journal.pone.0176879 28472177 PMC5417601

[31] Hsieh SY Hsu CY He JR Liu CL Lo SJ Chen YC. Identifying apoptosis-evasion proteins/pathways in human hepatoma cells via induction of cellular hormesis by UV irradiation. J Proteome Res (2009) 8:3977–86. 10.1021/pr900289g 19545154

[32] Liu H Wu X Dong Z Luo Z Zhao Z Xu Y. Fatty acid synthase causes drug resistance by inhibiting TNF-α and ceramide production. J Lipid Res (2013) 54:776–85. 10.1194/jlr.M033811 PMC3617951 23319743

[33] Madak-Erdogan Z Band S Zhao YC Smith BP Kulkoyluoglu-Cotul E Zuo Q. Free Fatty Acids Rewire Cancer Metabolism in Obesity-Associated Breast Cancer via Estrogen Receptor and mTOR Signaling. Cancer Res (2019) 79:2494–510. 10.1158/0008-5472.can-18-2849 30862719

