# Erratum: The Deubiquitinating Enzyme UCHL1 Induces Resistance to Doxorubicin in HER2+ Breast Cancer by Promoting Free Fatty Acid Synthesis

**DOI:** 10.3389/fonc.2021.687249

**Published:** 2021-04-14

**Authors:** Frontiers Production Office

**Affiliations:** Frontiers Media SA, Lausanne, Switzerland

**Keywords:** breast cancer, HER2+, chemoresistance, UCHL1, free fatty acid

Due to a production error, the email address for the corresponding author Wenjuan Wang was not correctly displayed. The correct email address for Wenjuan Wang is wangwenjuan1110@163.com. The publisher apologizes for this mistake.

The original version of this article has been updated.

