# Supplementary material for: The Deubiquitinating Enzyme UCHL1 Induces Resistance to Doxorubicin in HER2+ Breast Cancer by Promoting Free Fatty Acid Synthesis
Source: Front Oncol. 2021 Feb 24;11:629640. doi: 10.3389/fonc.2021.629640 (PMC7943833; doi:10.3389/fonc.2021.629640)
Supplement: Supplementary file 1 [file Table_1.docx]

**Supplementary Materials**

**Table S1.** Primers used for the real-time quantitative PCR

| **Primer name** | **Sequence (5′- 3′)** |
| --- | --- |
| human *UCHL1* | CCTGTGGCACAATCGGACTTA |
|  | CATCTACCCGACATTGGCCTT |
| human *GAPDH* | GGAAGATGGTGATGGGATT |
|  | GGATTTGGTCGTATTGGG |
| human *FASN* | AAGGACCTGTCTAGGTTTGATGC |
|  | TGGCTTCATAGGTGACTTCCA |
| human *ACACA* | ATGTCTGGCTTGCACCTAGTA |
|  | CCCCAAAGCGAGTAACAAATTCT |
| human *SREBF1* | ACAGTGACTTCCCTGGCCTAT |
|  | GCATGGACGGGTACATCTTCAA |

*UCHL1*, ubiquitin C-terminal hydrolase L1; *GAPDH*, glyceraldehyde-3-phosphate dehydrogenase; *FASN*, fatty acid synthase; *ACACA*, acetyl-CoA carboxylase alpha; *SREBF1*, sterol regulatory element binding transcription factor 1.
